# Observing Consistency in Online Communication Patterns for User Re-Identification

**DOI:** 10.1371/journal.pone.0166930

**Published:** 2016-12-05

**Authors:** Ikuesan Richard Adeyemi, Shukor Abd Razak, Mazleena Salleh, Hein S. Venter

**Affiliations:** 1 Information Assurance and Security Research Group, Faculty of Computing, Universiti Teknologi Malaysia, Skudai, Johor Bahru, Malaysia; 2 Information and Computer Security Architecture Research Group, Department of Computer Science, University of Pretoria, Lynnwood, South Africa; Universitat Rovira i Virgili, SPAIN

## Abstract

Comprehension of the statistical and structural mechanisms governing human dynamics in online interaction plays a pivotal role in online user identification, online profile development, and recommender systems. However, building a characteristic model of human dynamics on the Internet involves a complete analysis of the variations in human activity patterns, which is a complex process. This complexity is inherent in human dynamics and has not been extensively studied to reveal the structural composition of human behavior. A typical method of anatomizing such a complex system is viewing all independent interconnectivity that constitutes the complexity. An examination of the various dimensions of human communication pattern in online interactions is presented in this paper. The study employed reliable server-side web data from 31 known users to explore characteristics of human-driven communications. Various machine-learning techniques were explored. The results revealed that each individual exhibited a relatively consistent, unique behavioral signature and that the logistic regression model and model tree can be used to accurately distinguish online users. These results are applicable to one-to-one online user identification processes, insider misuse investigation processes, and online profiling in various areas.

## Introduction

Based on a series of empirical validations and several theoretical assumptions, the Internet is asserted to display fractal behavior with structural similarities irrespective of timescale. These self-similar characteristics observed on the Internet are partly attributable to the dynamic and uneven nature of individual activity patterns. Study on human dynamics on the Internet [1] can be attributed to the pioneering study in Barabasi [2] and Vazquez et al. [3]. In [2], it was observed that human dynamics can be replicated using the priority list queue probabilistic model. This model is based on the assumption that the length of the list of human tasks within a specified period, is a product of individual behavior. Human temporal characteristics are influenced by various factors, such as the task being executed, the constrained task completion duration, system and network inconsistencies, the individual’s sleeping patterns, environmental and seasonal changes, availability of resources, and the individual’s daily activity patterns [2]. These factors can be categorized based on the interests of the individual and the task requirements.

Zhou et al. [1] expounded on this logic using task- and interest-driven modeling processes. In a task-driven process, an individual is sandwiched between three different possible outcomes of the task execution: sequential, randomized, and priority execution patterns. In a sequential task, an individual executes the task in the order of the predefined sequence of arrival, which is either a last-in-first-out (LIFO) or first-in-first-out (FIFO) sequence. If such a task obeys a bounded distribution, the inter-execution time between tasks is homogenous. A randomized non-priority task, on the other hand, follows an exponential distribution with varying individual exponents. Priority-based task execution assumes that an individual executes a task in an order that is based on the highest priority factor, which does not consider the sequence of task arrival. Such a process tends toward a random probability with an exponential distribution (at probability P, P→0); otherwise, it is highly prioritized, which obeys a power law distribution (as P→1). However, in an interest-driven model, task execution varies based on the particular individual’s interest at time *t*.

Both task- and interest-driven models of human activity, are functions of the individual decision-making process [1]. This process is a complex phenomenon that requires the observation of various aspects of human navigation patterns and browsing characteristics [4] when measured on the Internet. The studies of these complexities have been limited, particularly in terms of the acquisition of reliable datasets on Internet interactions. The data collection process (invasive and non-invasive) is hampered by privacy, data insufficiency, and reliability. Other aspects of the exploration of human dynamics in online interactions are studies aimed at user attributions, such as web science and online profiling, and in more recent advances, online user reputation development [5,6], which are applicable for reliable and robust online object rating (an approximate judge of the quality of an object based on perception, usefulness and relevance). Web communication is an active research area that requires the development of a more complex model that can reveal deeper human behaviors.

In [7], for example, it is suggested that the Internet has permeated human day-to-day interactions, such that human unconscious behavioral signatures are semantically and syntactically observable on the Internet. Leveraging such signature in an online user identification process presents an unprecedented mitigation to various network security challenges, enhancement of multi-layer authentication processes in e-commerce, and the opening of research capacities in online communications. In this paper, we explored the various human dynamics that are based on the assertion that, if network burstiness is driven by human decision-making processes, and individuals exhibit varying characteristics of cognitive processes, then models can exist that could characterize individual online user, except when users share very similar behavioral and cognitive tendencies. Based on this assertion, this study attempts to address the following questions:

1) *How consistent are individual online communication patterns*? To address this question, the process through which non-invasive human-centric features can be extracted from online communication must first be addressed. Research findings in [7–9] have established that humans exhibit varying patterns in online communications when aggregated over a given period. It is, therefore, logical to posit that humans exhibit relatively stable behavior when they are not the subjects of observation. 2) If humans exhibit relatively stable behavioral tendencies, *is it sufficient to reliably distinguish individuals on the Internet*? To the best of our knowledge, this study marks the first attempt to explore individual behavioral consistency in online communications based on human tempo-spatial characteristics.

The remainder of this paper is organized as follows. In the next section, the underlying assumptions and theories on which the individual user is observed are presented. In addition, related works on human web interactions are also discussed. In the ‘Dataset and Preprocessing’ section, the dataset used in the study is explained. The section ‘Exploration of Behavioral Dynamics’ presents the empirical observation, analysis, and discussion of the observed individual behavior. The observations deduced from the empirical process conducted in this study, recommendations, and the limitations of the study are presented in the section termed ‘Reliability of Observed Patterns’. The summary of the study and the findings, are further presented in the ‘Conclusion’ section.

## Theory of Individual Dynamics

Network traffic modeling process is fundamentally assumed to be highly stochastic, independent, and occurring at a constant rate. This assumption was initially believed to obey the Poissonian model distribution [2]. However, empirical findings have refuted this assumption in favor of a tailed distribution [3], which obeys the power law of the form *P*(*t*) ≈ T^-α^.

The exponent value (also referred to as scaling factor), α, of value -1 and 3/2 can be used to mimic human communication patterns. Furthermore, Barabasi [2]observed that the priority-list queuing mechanism of the form

$$P(\tau )=\rho (\tau^{-1/\gamma })/\tau^{\left(l+\delta \right)/\gamma }\;for\;\{\gamma \to \infty ,\;l\to based\;on\;highest\;priority\}$$ (1)

can be used to model the inter-event time of human communications (e-mail, in this case). Similarly, Vazquez et al. [3] observed that the power law distribution can be used to explain human browsing patterns. They maintained that both human browsing and e-mail communication obey the power law with a universal class of α = 1. Zhou et al. [1] shared a similar assertion except that, the exponent α, was not universal. The study revealed that, although the inter-event times and response rates of e-mail communication, short message communication, web browsing, and movie watching adheres to non-Poissonian statistical characteristics that are appropriately modeled by a power law, they all show varying exponents, as depicted in Table 1.

**Table 1:** Correlation between Models of Inter-Event and Response Times.

Event	Recorded exponent (α)	Individual Basis
E-mail	-1	Slight variation
SMS	-1.52 to -1.7	Correlation between average number of message per day and α
Web browsing	-2.1 to -3	Variation
Movie watching	-2.08	Slight variation

The study further asserted that human dynamics could be modeled with an interest-driven model that satisfies the principle of diminishing return and the converse, using the following:

$$\lambda (t)=\frac{a}{t}\int_{0}^{t}dt\lambda (t)$$ (2)

In addition, Dezso et al. [10] observed that online news website visitation patterns decay in power law distribution. Visitation pattern refers to the general visit characteristics of users. These include the visitation of news document, web page visitation history, and cumulative web page visitation. The study posits that the interval between consecutive HTML requests by an individual is uneven and can be suitably modeled with the power law of exponents, α ≈ 1.2 ±0.1. A general consensus exists on a suitable model for human activity patterns—that is, the power law, but with varying exponents. In [4], it was observed that browsing patterns can be generally modeled using a log-polynomial exponential function of the form

$$f(x;\theta )=exp[\sum_{i=0}^{n}\theta_{i}\;\log{\left(x\right)}^{i}]$$ (3)

Through analysis of human activity patterns, the study showed that the model

$$f(x)=exp[-0.056\;\log x^{2}-0.26\;\log x+10.15]$$ (4)

efficiently captures the nonlinearity of human dynamics, which is beyond the capability of the conventional power law. The study, thus, asserted that the adoption of the power law in human dynamics modeling might display systematic deviations. It is worthwhile to note that the identified studies on human dynamics are targeted at generically establishing a model of human behavior. Such a generalization is useful in network traffic measurements. However, it is limited in terms of explicit individual applicability in areas such as web personalization and user identification, which are domains that require explicit individualization. Examples of the systematic loss of individualization in an attempt to derive a generic model fit are provided in the ‘Exploration of Behavioral Dynamics’ section of this study.

It should be further noted that the derived log-polynomial exponential function in [4] is another representation of power law without the systematic deviation and is depicted as follows:

$$f(x)=exp[-0.056\;\log x^{2}-0.26\;\log x+10.15]\equiv 25591x^{-0.372}$$ (5)

In essence, the generation of power law or the log-polynomial function describes individual characteristic features of inter-event times. This study thus attempts to explore the dimensions of individual patterns in terms of possible user signature derivations. Similar to [10], wherein user visitation patterns were explored, this study explored the dimensions of knowledge worker visitation patterns, as well as the structural distinction underlying individual web request patterns. Similarly, as asserted in [5,9,10], this study further suggest that human web browsing behavior contains unconscious behavioral patterns that are unique to the individual. In [8], the existence of digital ‘click prints’ in online communication patterns was observed. Similar assertions were provided in [7][11].The term click print was coined to generally refer to a unique and distinguishable consistent behavioral pattern, observed in the browsing pattern of a user.

The present study focuses on the assertion that, if human cognitive processes drive human bursty nature on the Internet, and humans demonstrate varying level of cognitive disposition, individual web patterns can be distinguished, except when the individuals under observation, exhibit relatively similar cognitive processes with another individual. To test this assertion, a server-side web traffic dataset was collected from the Research Management Centre (RMC) of a research university in Malaysia. Details of the dataset used, the method of data cleaning and analysis are presented in the subsequent sections.

## Dataset and Preprocessing

A major limitation in the comprehension of human complexity on the Internet is the unavailability of a reliable human-centric dataset. Human-centric network data can be captured at either the client side (such as a web browser) or server side, or both. The process of capturing can be invasive or non-invasive. An invasive methodology requires the installation of a network scanner or a capturing tool or script on the capturing system. This method can capture a relatively higher volume of human activity, which is subject to diverse privacy and security concerns. Additionally, it is highly dependent on an agreement with the user. A non-invasive methodology, on the other hand, may not capture a huge volume of human activity, but it presents less of a privacy or security risk. A discussion of the advantages, limitations, and structure of data collection methodologies is found in [4]. In the present study, we adopted a non-invasive server-side data collection methodology. Description of the data collection process and the preprocessing is presented in the proceeding sections.

### Server-side Data Collection

Having obtained formal approval from the RMC ethics board on research activities, a user-request-capture script was used to log the activity of every RMC user at the Universiti Teknologi Malaysia between April 26, 2014, and September 22, 2014. The human-initiated request log record format included the date and time of each request, the login name of the user, and the URL of the requested page. Although a typical log record contains several fields, in this study, we captured only key fields that are human-centric. Individual user requests were saved as a separate request dataset such that the activity pattern of each user was logged 24 hours daily. The users in this study were staff members of the RMC. To recruit users (respondents) for this study, a consent form was distributed to all RMC staff members. Sixty-four respondents completed the consent form, indicating their willingness to participate in the study.

The RMC is comprised of five departments that manage the daily research activities of the university. The research activity is hosted on a web portal that employs a load balancing architecture consisting of two web servers. These Servers provide intensive web services and documents, which are designed in four web Server-modules, each consisting of several sub-modules and sub-sub-modules. Although the data-capturing period spanned several months, the daily data pattern per user was not the same, as the activity period of the user is not preprogrammed or stationary. Some users within the collection period went on leave, training, or holiday. Furthermore, network downtime also occurred. Additionally, the data was collected with the assumption that the web cache and web proxy did not affect individual requests to the Servers or the user capability of performing write operations (URL requests) to the Servers. However, if these were affected, the estimated margin would be negligible [12]. Individual respondents and users are herein used interchangeably to refer to those under observation. The server-side data collected for this study does not contain any external network traffic source or network traffic from any other server, apart from the RMC servers.

### Data Preprocessing

**Fig 1:**
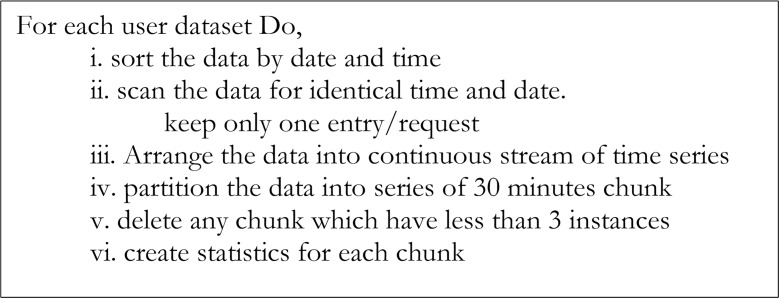
Session creation Algorithm.

The data collected from the servers contained repeated user actions, which could be the result of the browser refreshing or the effect of the network relay. To minimize the probable error, data cleansing was carried out on the Server-side data. In [12] and [13], it was observed that the typical inter-request time of human-generated web traffic varies between 1 second to 2 seconds. It is logically inconceivable for a user to request a given URL in milliseconds; thus, a benchmark of the inter-request time of 1 second was adopted as the minimum interval between two probable consecutive requests. A heuristic process was developed for the preprocessing of the dataset, which involved data cleansing and creating web-browsing sessions of user requests. The duration of browsing varied among users. In [10], a session duration baseline of 1 hour was adopted, whereas the session duration from empirical observation in [14] was approximated at 25.5 minutes. In [15], it was observed through an empirical investigation using a log-log complementary distribution that a browsing session can be bounded with 10,000 s (166.66 minutes). However, a session delimiter of 30 minutes is commonly used [16] in the sessionization of web browsing pattern. The session timeout defined in [16] is based on inactivity ≥30 minutes. However, this logic is not applicable in a workplace where systemic time structure is constantly followed. Systemic time structure depicts a regular time routine; such as 8:00am to 5:00pm work hours, as used in workplaces. Such inactivity-logic could limit the observed pattern to the type of work, and the schedule of the individual, which may not necessarily reflect the pattern of the user. The current study, therefore, considers the logic of 30 minutes session boundary that can provide a logical benchmark, based on the assumption that a human generally relaxes after 30 minutes of continuous work. It additionally assumes that the individual is believed to be working on a given task, which is not based on browsing for interest. This can be illustrated, for example, between two individuals, A and B. Individual A is given a workload of a task executable on the Internet within a set duration. Depending on the motivation, strength, and availability of resources, Individual A may continuously work for a long period. However, the working pattern will follow a constant trend with a possible intermittent break until task completion. The use of a session timeout can enable measurement of the capability of Individual A. In contrast, for Individual B, tasks are executed without a planned action or the ending of a task. Obviously, the choice of the performance metric for each individual will differ. As highlighted in [17], a session timeout can be defined through training with a combination of predictors of queue properties. However, for the sake of uniformity, research replicability, and future comparison, we adopted the 30 minutes session boundary, which is not based on inactivity but on consecutive periodic activity. The 30 minutes session implies that the individual dataset is partitioned into a continuous delineation of 30 minutes irrespective of the level of activity. For instance, if a user worked continuously from 8:00am to 9:23am and then resumes for the rest of the day from 1:15pm till 3:10pm, a total of seven sessions (8:00am-8:29am, 8:30am-8:59am, 9:00am-9:23am, 1:15pm-1:44pm, 1:45pm-2:14pm, 2:15pm-2:44pm, and 2:25pm-3:10pm) will be recorded for that day, for such a user. Therefore, a session is defined in this study to mean an active period not exceeding a 30 minutes boundary. A snippet of the algorithm for the session creation process is presented in Fig 1. Furthermore, this boundary duration provides more granular abstraction than a greater duration.

### Feature Extraction

The heuristic output generated a user click-stream data: an ordered list of all web pages viewed by each user. Because we are concerned with the analysis of individual dynamics, we considered features that depend on individual behavior. The integration of more human-centric features in the online user identification process is one of the distinctions between this study and existing studies. Ordered moments, such as the mean, standard deviation, skewness, kurtosis, and variance of dispersion, were extracted, as discussed in [18]. Furthermore, visitation patterns were also considered. Individual visitation patterns account for page visitation characteristics, including aggregated visitation patterns, rate of visits per session, and the session length structure of the user’s request within the bounded URL under observation. The definition of user interest is arguably equivalent to the task priority as asserted in [10]. This study assumed that the task-priority execution is subject to individual disposition among other factors [19]. The visitation pattern is similar to the observation in [13], wherein user visitation patterns were observed within and across sessions. The present study slightly differs in that, it considers visitation patterns within sessions and aggregated visitation patterns within the duration of the observation, without repetition of visited URLs. The URLs in all modules of the RMC Servers are given by the total number of possible unique URLs in the two Servers as denoted by

$$U_{m}=\sum_{i=1}^{N}{URL}_{i}$$ (6)

The study further assumed (in line with the empirical findings in Eq 5) that the rate of requests obeys a power distribution given by

$$R(t)\approx r^{-\alpha }$$ (7)

where α assumes a continuous data format. Aggregated visitation patterns (*V*_*agg*_) within a session, the rate of revisits per session (*R*_*vs*_), and session length based on aggregated visits (*S*_*agg*_) are given by Eqs (8), (9) and (10) respectively. Aggregation is adapted as defined in [9].

Vagg=∑i=1n(URLpersession)i∑jN(URLunderobservation)jwheren⊂N,andi⊂j (8)

Rvs=∑i=1n(URLpersession)iSdSd⟹sessionduration=∫j=1ntjdt,≤30minutes (9)

Sagg=∑jN(URLunderobservation)jSd (10)

The extracted features can be classified into session characteristics (including the total number of requests in a session and the duration of the session), request characteristics (including the inter-request time series and ordered moments of flights and intervals), and visitation characteristics (which include aggregated visitation patterns, the rate of visitations per session and aggregated visitation patterns per session length). Summary of the extracted features is presented in Table 2:

**Table 2:** Descriptive summary of the extracted features.

Features Used	Label	Brief Description
Aggregated visitation pattern	(*V*_*agg*_) {f1}	It is the ratio of the sum of the total URLs visited in a session to the sum of URL-count (URL under observation) in the session. URL-count refers to the sum of the number of times any URL is revisited within the duration of a session. It also shows the sequential/parallel characteristics of the individual. This feature reveals the degree of linearity in online browsing behavior.
Rate of visits per session	(*R*_*vs*_) {f2}	It is the ratio of the sum of the total number of URLs visited in the session, to the duration of the session. This feature shows the visit behavior of an individual within a session.
Rate of visit-count per session	(*S*_*agg*_) {f3}	It is the ratio of the sum of URL-count to the duration of the session. This feature shows the re-visitation behavior of an individual within the duration of a session.
Total number of requests per session	{f4}	It is the total number of requests made within the duration of a session. This feature shows the behavior of an individual with respect to the amount of request capacity. It also indicates the nature of the task being handled by the individual.
Session duration	{f5}	It is the absolute difference between the end time of the session and the start time of the session. This feature reflects the behavior of the user within the delimited session of 30-minutes.
Interval and Flight Mean	{f6 and f7}	The mean of a distribution reveals the standard shape parameter of individual request pattern over the observed duration.
Interval and Flight Standard Deviation	{f8 & f9}	The standard deviation of a distribution reveals the degree of spread-out of individual request pattern within the period of observation. This feature will reveal the inherent work pattern of each user.
Interval and Flight variance	{f10 & f11}	The variance of a distribution is similar to the standard deviation distribution. It measures the degree of proximity of individual request pattern over the period of observation.
Interval and Flight Skewness	{f12 & f13}	The skewness of Interval and Flight measures the degree of asymmetry of individual request pattern within the period of observation.
Interval and Flight Kurtosis	{f14 & f15}	These features show the behavior of individual request pattern based on its peak width and tail weight. They also measure the degree of an outlier in request pattern.

In the next section, we present the results of the various exploratory processes of the online user identification process.

## Exploration of Behavioral Dynamics

The aim of this study is to reveal the inherent (dis)similarity in online users for online identification purposes. To distinguish individuals, it is logical to investigate individual consistency in online communications. Consistency in this context is defined as a strong similarity in the communication patterns of individuals through a defined interval of observation. It is supported by the assumption in the principle of cognitive consistency [20], which states that humans desire consistency in their beliefs, attitudes, and behaviors and that dissonance motivates efforts to achieve consistency. To assert the consistency in user online communications, we collected a seasonal Server-side dataset of known users. The season centers around the Ramadan fasting period (pre-, during, and post-fasting), as shown in Table 3, with consideration of the seasonal influence being sufficient to reveal any pattern inconsistencies. Periods ‘a,’ ‘b,’ and ‘c’ respectively represent the period prior to the fasting season, the fasting season, which spans one month, and the period after the completion of the fasting exercise respectively. A combination of the seasonal traffic was then explored.

**Table 3:** Seasonal Duration for Consistency Exploration.

Season	Duration
a. Pre-fasting period	April 24 –June 30, 2014
b. Fasting period	July 1 –August 1, 2014
c. Post-fasting period	August 2 –September 2, 2014

Server-side data of 60 respondents were collected for the study. However, given the substantial volume of the data, a sampling method was applied. In [9], it was observed, through empirical observation, that an aggregation of ≥ 300 online user sessions is sufficient for an effective online identification process. Thus, 300 sessions were adopted as the baseline sample for each user. In addition, 5,000 inter-request numbers were added to the baseline metrics. The choice of 5000 was based on the distribution of the request pattern of the sampled users, and the need to generate a substantial reliable sample of users for the study. Inter-request is defined in this study to mean the interval between two successive requests, such that for every given request number (*R*_*n*_), there is a corresponding number of inter-requests (*R*_*n*−1_). Traffic patterns of 11 known users met these criteria. Table 4 gives the summary of the requests and sessions of each user. The distribution of sessions and request sizes for each season did not follow any obvious pattern, which is expected because the durations of the seasons were not uniform. Furthermore, the nature of the task and daily activity patterns in terms of the number of requested URLs from the Servers may not always be exactly the same.

**Table 4:** Summary of User Requests and Sessions.

Users	Season	Number of Requests	Number of Sessions
1	a	6,544	268
	b	2,408	110
	c	9,944	297
2	a	6,107	320
	b	1,437	94
	c	915	71
3	a	9,215	365
	b	7,198	176
	c	17,662	364
4	a	3,101	186
	b	1,382	77
	c	1,392	109
5	a	3,739	200
	b	2,086	143
	c	2,729	181
6	a	7,584	223
	b	3,109	102
	c	4,728	140
7	a	7,035	288
	b	3,194	122
	c	4,335	218
8	a	12,456	417
	b	4,568	159
	c	10,569	294
9	a	2,728	155
	b	1,162	75
	c	2,710	204
10	a	5,717	253
	b	2,726	128
	c	4,723	197
11	a	2,751	156
	b	2,183	71
	c	10,569	294

Because the aim of this study is to observe the probability of consistent behavior in individual online communication patterns, we define the unit of online communication to be the request. A request is initiated by a user through web click, typed URL or any action from the user that resulted in the eventual communication between the user client (web browser) and the server. A typical client-server communication is predicated on the request-response model. The response of the server is subject to network conditions, applications, and system factors, whereas the user request is primarily dependent on the user. Using the inter-request time as the unit of measurement, time-series data were therefore extracted from the sessions of each individual, based on the seasonal distribution. In the next section, a detailed description of the process used to extract patterns from individual clickstream data is presented.

### Transformation of Inter-request Patterns

Textual transformation technique is often applied to time-series data to reveal recurring patterns that are useful sub-sequences of the original time-series data. A more recent tool was presented in [21], which is an extensive improvement to the bag-of-pattern representation in [22]. This tool uses the principle of symbolic aggregate approximation (SAX), which was initially developed based on findings in [23], to extract variable-length recurring patterns as well as signature patterns from time series. The technique explores SAX for dimension reduction and discretization while implementing a sequitur and linear spatiotemporal algorithm that can reveal a context-free relationship from a given string. This tool simply extracts the common sub-sequence observed in a given time series using rule-based notation, such that the common sub-sequence on local and global semantics are detected. The SAX parameter-sliding window length (*ω*), piecewise aggregate approximation (*η*), and alphabet size (*α*) optimization were initially performed on the data. As suggested in [22,23], a smaller value of *ω* and somewhat larger value of *η* can be adapted to a relatively smooth time series. Time-series transformation can be carried out with different combinations of the SAX parameter. However, from the initial experimental processes carried out, it was observed that the following combinations *ω* = 32, *η* = 4, and *α* = 6, provided relatively stable common subsequences. Table 5 gives the summary of the number of rules generated for each season for each user.

**Table 5:** Revealed Consistency in Individual Request Patterns.

User	Number of observed rules per season			Total number of common rules in all seasons
	a	b	c	
*1*	288	117	472	45
*2*	267	68	41	16
*3*	398	352	841	109
*4*	192	90	70	15
*5*	213	128	141	26
*6*	390	170	255	55
*7*	321	153	203	45
*8*	615	246	518	103
*9*	124	55	111	9
*10*	304	144	239	41
*11*	124	116	518	34

All common sub-sequences ($acs=\sum_{r=1}^{n}{ss}_{r}\;,\;where\;{ss}_{r}=$ rule-generated based on sub-sequence) were extracted for each user, with result as presented in Table 5. The number of consistent rules is defined by the expression

$$[\left\{a\cap b\cap c\}\subseteq \{a\cup b\cup c\right\}]\in Q,\;where\;Q\;is\;the\;possible\;combination\;of\alpha^{\omega }pattern\;sizes$$

The expression indicates that the total number of consistent rules is the intersection among the three seasons under observation. Rules in this sense are described as a reoccurring pattern inherent in the inter-request time of each user. It was observed that each user exhibited consistent patterns across all the seasons. As expected, users with averagely higher frequencies of request pattern, which culminated into a higher number of observed rules, demonstrated higher numbers of consistent rules over the duration of observation. This observation supports the theory of cognitive consistent behavior previously mentioned. Moreover, SAX presents a context-independent pattern-exploration process. Human behavior viewed in a continuous span of behavior reveals a more probable behavioral consistency measure; hence, the need for context-dependent human behavior is likewise revealed. Such behavior can disclose a semantic relationship in individual communication patterns over a given period. In the next section, the pattern exploration of the individual user is discussed.

### Individual Pattern Modeling

**Fig 2:**
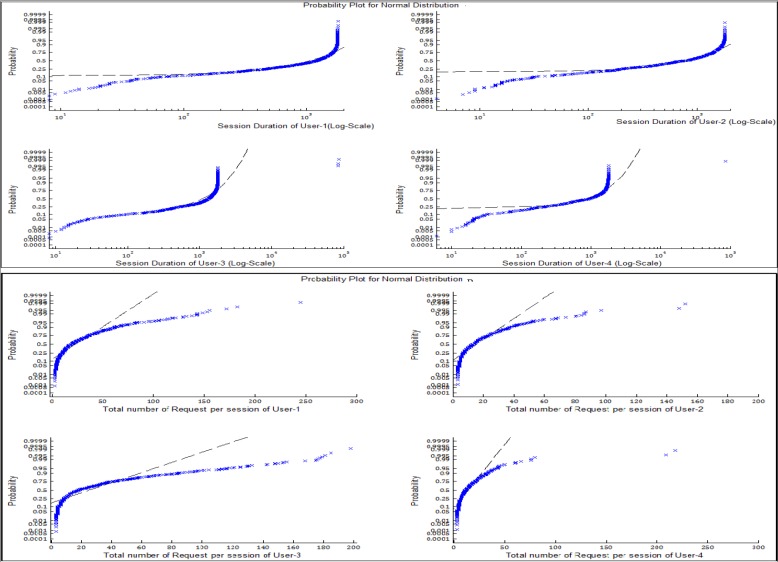
Cumulative Probability Plot of Session Characteristics for Four Users.

The exploration of habitual user patterns on electronic media, such as the Internet, comprises complexities that can be segmented into the various fundamental forms of human nature. Visual observation of the individual session characteristics—the total number of requests in a session, and the session duration, as shown in Fig 2—reveals that an individual exhibits a pattern that slightly differs from other users. This observation thus supports the notion of the probability of the existence of a unique behavioral pattern for each user. In this section, we explore visitation characteristics of each individual with respect to session duration. This is to further understand the compositional features that can be used to explore the observed probability in Fig 2.

Fig 2 depicts a high level of user abstraction. To further understand the correlations in browsing patterns of each individual, exploration of visitation patterns of each user with respect to session duration was performed. Tables 6 and 7 provide the model fit and parameter for each user.

**Table 6:** Model of Session Duration for Visitation Pattern Aggregation per Session.

User	Model fit parameter					
	Power law		Polynomial law		Exponential law	
	Model	R^2^	Model	R^2^	Model	R^2^
*1*	1962*x*^−1^	1	4*e*^−4^*x*^2^ − 0.9781*x* + 525.32	0.461	193.81*e*^−0.002*x*^	0.798
*2*	9057*x*^−1^	1	3*e*^−4^*x*^2^ − 0.6523*x* + 313.66	0.381	109.19*e*^−0.002*x*^	0.762
*3*	19981*x*^−1^	1	3*e*^−6^*x*^2^ − 0.2637*x* + 396.54	0.296	$30.009e^{-8e^{-5}x}$	0.119
*4*	6294*x*^−1^	1	1*e*^−6^*x*^2^ − 0.0935*x* + 134.55	0.297	$14.52e^{-1e^{-4}x}$	0.095
*5*	9121*x*^−1^	1	2*e*^−4^*x*^2^ − 0.518*x* + 259.67	0.366	114.85*e*^−0.002*x*^	0.820
*6*	1539*x*^−1^	1	4*e*^−4^*x*^2^ − 0.9235*x* + 461.07	0.503	200.8*e*^0.002*x*^	0.805
*7*	15275*x*^−1^	1	2*e*^−6^*x*^2^ − 0.2053*x* + 291.1	0.266	$32.513e^{-9e^{-5}x}$	0.101
*8*	28568*x*^−1^	1	4*e*^−6^*x*^2^ − 0.35*x* + 531.58	0.234	$45.009e^{-9e^{-5}x}$	0.106
*9*	7187*x*^−1^	1	2*e*^−6^*x*^2^ − 0.184*x* + 231.48	0.183	$25.229e^{-1e^{-4}x}$	0.084
*10*	13792*x*^−1^	1	2*e*^−6^*x*^2^ − 0.2037*x* + 295.01	0.280	$27.022e^{-1e^{-4}x}$	0.093
*11*	8718*x*^−1^	1	3*e*^−6^*x*^2^ − 0.2347*x* + 320.48	0.184	$34.59e^{-1e^{-4}x}$	0.088

**Table 7:** Model of Number of Requests per Session for Aggregated Visitation Patterns.

User	Model fit parameter					
	Power law		Polynomial law		Exponential law	
	Model	R^2^	Model	R^2^	Model	R^2^
*1*	5*e*^−5^*x*	1	4*e*^−21^*x*^2^ + 5*e*^−5^*x* + 1*e*^−17^	1	5*e*^−4^*e*^0.0268*x*^	0.740
*2*	**1*e***^**−4**^***x***	1	2*e*^−20^*x*^2^ − 1*e*^−4^*x* + 2*e*^−17^	1	7*e*^−4^*e*^0.0395*x*^	0.747
*3*	5*e*^−5^*x*	1	1*e*^−21^*x*^2^ + 5*e*^−5^*x* − 2*e*^−17^	1	4*e*^−4^*e*^0.0249*x*^	0.763
*4*	2*e*^−4^*x*	1	1*e*^−20^*x*^2^ + 2*e*^−4^*x* − 3*e*^−17^	1	1.1*e*^−4^*e*^0.0321*x*^	0.603
*5*	**1*e***^**−4**^***x***	1	−4*e*^−20^*x*^2^ + 1*e*^−4^*x* − 4*e*^−17^	1	6*e*^−4^*e*^0.0505*x*^	0.8034
*6*	6*e*^−5^*x*	1	−6*e*^−12^*x*^2^ + 6*e*^−5^*x* + 4*e*^−8^	1	5*e*^−4^*e*^0.0284*x*^	0.7861
*7*	7*e*^−5^*x*	1	4*e*^−20^*x*^2^ + 7*e*^−5^*x* + 4*e*^−17^	1	4*e*^−4^*e*^0.0369*x*^	0.7924
*8*	4*e*^−5^*x*	1	1*e*^−20^*x*^2^ + 4*e*^−5^*x* + 5*e*^−17^	1	3*e*^−4^*e*^0.0264*x*^	0.7446
*9*	**1*e***^**−4**^***x***	1	−5*e*^−20^*x*^2^ + 1*e*^−4^*x* − 4*e*^−17^	1	7*e*^−4^*e*^0.0475*x*^	0.7696
*10*	7*e*^−5^*x*	1	3*e*^−20^*x*^2^ + 7*e*^−5^*x* + 3*e*^−17^	1	4*e*^−4^*e*^0.0434*x*^	0.8501
*11*	**1*e***^**−4**^***x***	1	2*e*^−21^*x*^2^ + 1*e*^−4^*x* − 8*e*^−18^	1	8*e*^−4^*e*^0.0248*x*^	0.7148

The power law model best fit the behavior of all users in each observation. The session duration of the visitation patterns of observed individuals shown in Table 6 reveals a consistent power law distribution with a varying coefficient. The observed coefficient reveals a dissimilar model parameter for each user, which indicates the tendency of a unique and consistent pattern of the visitation duration within the defined session delimiter of 30 minutes. In essence, given the duration of any given user within a 30-minutes defined session, the visitation pattern of such a user can be inferred. The result in Table 6 also reveals that the power law expression accurately mimics the session duration for each User, as shown by the model fitness (R^2^ = 1).

In addition to the power law distribution, the number of requests per session shown in Table 7 followed a polynomial distribution. The observed polynomial model is a generalized form of the power law model. For instance, the power law expression for user 1 (5*e*^−5^*x*) is a subset of the polynomial law expression given as; 4*e*^−21^*x*^2^ + 5*e*^−5^*x* + 1*e*^−17^. The measure of model fitness (R^2^ = 1) for both power law and polynomial law, shows that the model perfectly mimics the number of requests for each user. However, the model fitness for the exponential law (R^2^<1) shows that the exponential law cannot perfectly mimic the number of requests of each individual. The model coefficient of the power law model in Table 7 reveals that different users can be represented by the same model coefficient and scaling factor (for example, User-2, User-5, User-9, and User-11 have a coefficient of **1*e***^**−4**^) based on the pattern of the number of requests per session. This observation is similar to that found in [1,4], where online behavior is asserted to obey the power law. However, as evident in Tables 6 and 7, multiple users can have similar model fit parameters. While this observation subtly implies different human behaviors in online interactions, it does not provide a significantly unique distinction for the online user identification process. Thus, as addressed in the following section, this study further explored the probability of online user distinction based on the structural relationship among all features. Machine-learning models are suitable for exploration of such characteristics. To ensure maximum data representation and adequate sessions for the machine-learning exploration process, the duration of data collection from the RMC servers was extended to December 31, 2014. The next section discusses the application of machine learning technique to observe the (dis)similarities among the users.

### Exploration Based on Individual Cluster Patterns

Clustering is an unsupervised machine-learning technique that does not rely on a predefined supervision or input from a supervisory agent. It is the process of discovering subsets of a dataset that have relatively similar patterns among themselves but relatively dissimilar patterns compared to other subsets. Distance within similar subsets is referred to as intra-cluster distance, while the distance between dissimilar subsets is referred to as inter-cluster distance. The aim of clustering is to minimize the intra-cluster distance while defining a maximum boundary between dissimilar subsets. Clustering can be hierarchical, partitioning, or conceptual. The partitioning process involves partitioning the data space into *n* subsets; the hierarchical group subsets are extracted based on hierarchical decomposition [24,25]. The conceptual clustering process can be hierarchical in nature, but with a more logical assumption, as discussed in [26].

**Fig 3:**
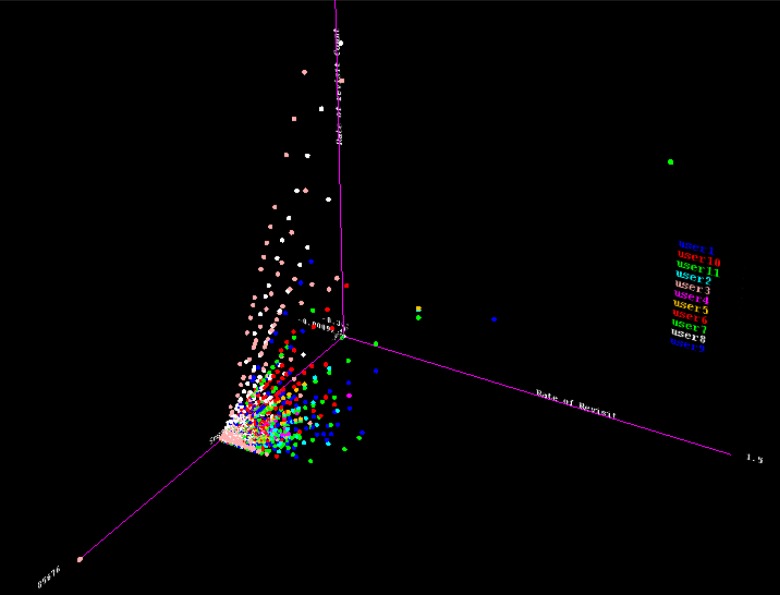
3-D Plot of Experimental Data.

Table 8 presents the results obtained from various clustering algorithms using the WEKA 3.7.11 workbench [27], which was developed at the University of Waikato and implemented in Java. WEKA is an open source tool that has been well adopted for research studies on machine learning and artificial intelligence in general. The input data is preprocessed using the features discussed in the ‘Dataset and Preprocessing’ section. These features include session characteristics (session length and the total number of requests in a session), visitation patterns (visitation rates, revisitation rates, and the rates of revisitation counts), and request characteristics (first, second, third, and fourth ordered moments as discussed in [18]). 3D plots of the revisitation rates, rates of revisitation counts, and session durations in the x, y, and z-axes, respectively, are presented in Fig 3. The data in the figure is characterized by a shapeless, inseparable-boundary hyperplane, such that the probability generating a discrimination boundary is significantly low.

**Table 8:** Cluster Evaluation on a Class of 11 Users.

Number of classes	Percentage of accuracy for cluster to class evaluation							
	EM	Cobweb	Hierarchical	Canopy	SOM	Density-base	k-Means	LVQ
11	13.70%	14.05%	14.31%	14.39%	14.82%	13.67%	13.70%	15.07%

The clustering result in Table 8 further supports the assertion of a poor cluster boundary; that is, a linearly and nonlinearly inseparable class boundary among online user patterns. A total of eight clustering algorithms were explored based on class-to-cluster the evaluation measure. The class-to-cluster measure provides a measure of visualizing the probable class that was accurately classified. The explored clustering algorithms include expectation maximization (EM), Cobweb, hierarchical, canopy, self-organizing map (SOM), density-based, *k*-means, and learning vector quantization (LVQ), as shown in Table 8. EM is a probabilistic model that calculates the likelihood estimate of the measurement parameter. It statistically depends on unobserved latent variables through the log-likelihood function and computes parameter maximization. This approach, as shown in Table 8, is not suitable for inter-cluster discrimination of the observed data. Cobweb clustering is a conceptual approach that incrementally navigates the instance space to create a tree-like structural boundary whose leaves represent individual concepts, branches depict a hierarchical cluster, and root node represents the data space [26,28].

Cobweb clustering defines the boundary based on the intrinsic and interactive characteristics of the instance space. The hierarchical clustering algorithm merges data points based on a lesser dissimilarity index to form a single cluster. The hierarchical cluster algorithm in WEKA employs agglomerative hierarchical pattern discovery with the capability of different distance measures. A hierarchical algorithm depends on the previously identified clusters. For a shapeless boundary of online user patterns, such a paradigm limits the clustering capability. The canopy clustering algorithm (overlapping subset of instance spaces) uses loose distance (*t*_1_) and tight distance (*t*_2_) density threshold heuristics (where *t*_1_ < 0 implies a positive multiplier for *t*_2_) to define the cluster boundary [29]. The heuristics are based on the standard deviation of the attribute of the instance space. The SOM technique works as an intermediary to effectively identify discriminant features in a dataset. It creates a prototype for data representation by keeping the topological projection of the created prototype through mapping of d-dimensional input to the low-dimensional grid. It uses the inherent data structure to minimize the intra-cluster distance and maximize the inter-cluster distance. The density-based clustering algorithm attempts to find a nonlinear shape structure in instance space by computing the density properties. WEKA implements a ‘make density-based’ clustering algorithm that employs the wrapper approach. It uses density reachability and a density connectivity line to define the cluster boundary. The *k*-means clustering algorithm is a partitioning algorithm that iteratively classifies data into *k* clusters by the distance measure until a local minimum criterion is satisfied. Similarly, LVQ uses the distance measure. It employs neural network structure clustering to establish the boundary by approximating the prototype distance. The cluster-class decision is based on the ‘winner takes it all’ scheme [30].

The results from the eight cluster algorithms with a modal accuracy of 15.07%, *μ* = 14.22%, and *σ* = 0.54 lend insight to the structural complexity and correlations in online user patterns. The performance of these algorithms can be attributed to boundary discriminant formation and structure decomposition. In the next section, supervised machine-learning techniques are explored to reveal probable smaller intra-user patterns and larger inter-user patterns.

### Exploration Based on Classification of Individual User

A survey on various supervised machine-learning methods on Internet traffic classification [25] has revealed that various classifiers have been applied to classification problems in network settings. These include a *k*-nearest neighbor, linear discriminate analysis, quadratic discriminant analysis, fast correlation-based filter, genetic algorithm, generalized naive Bayes (kernel estimate), J48 decision tree, and support vector machine. These classifiers can be generally categorized into the linear discriminant class description, nonlinear discriminant class estimation by the projection/kernel method, rule-based class formation, and the ensemble learning process. This study explores all identified categories of classifiers to reveal the probability of individual distinctions in online interactions (detail discussion of various machine-learning algorithms can be found in [31] and [32]).

**Fig 4:**
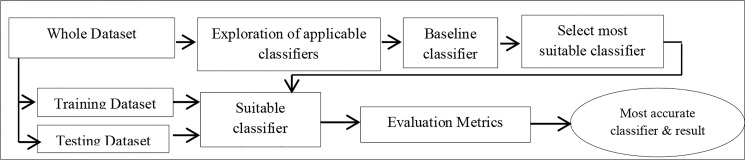
The process of Classification.

An experimental process of classification was conducted on the dataset comprising 11 sampled users, as discussed in ‘Exploration of Behavioral Dynamics,’ using WEKA toolkit. Studies in [7,31] assert that WEKA lends itself to convenience and ease of automation within the script and was thus the choice for classification in this study. Fig 4 illustrates the experimental process employed for this section.

The experiment was conducted in two phases. The experimental process for a diverse range of classifiers was initially performed, as shown in Table 8. We then leveraged the results in Table 8 to select classifiers with a relatively higher accuracy with respect to the baseline classifier criteria. The selected classifiers were adapted for the next phase, as illustrated in Fig 4. The study explored 22 different classifiers with the aim of investigating the probability of distinct feature correlations that can distinguish patterns among the selected users. The experimental process was based on ten-repetition ten-fold cross validation with a p-value of 0.01. To evaluate the performance of each classifier, seven evaluation metrics were considered, as shown in Table 9. Classifier accuracy describes the degree of difference between the correctly classified (true-positive and true-negative) instance and the actual instance. The root mean square error (RMSE) measures the magnified difference between the correctly classified instances and actual instances. RMSE (the order of importance values range from 0→1) is biased toward larger errors. This characteristic makes it suitable for prediction performance evaluation. The precision of a classifier (0→1) computes the ratio of correctness over the classified instances. It describes the consistency of the classifier. Recall (0→1) evaluates the performance of a classifier based on the probability of the correctly classified instance. The area under the curve (0→1), which is also referred to as the receiver operating characteristic curve, is the cumulative distribution function (CDF) of the true positive (TP) to the CDF of the false positive (FP).

**Table 9:** Performance Evaluation of Explored Classifiers.

Supervised ML Taxonomy	Metrics / Scheme	*Accuracy* (%)	*Kappa Stats*	*RMSE*	*Precision*	*Recall*	*F-measure*	*AUC*
Tree/Logic-based	*ZeroR (Baseline)*	14.42(0.07)	0.00(0.00)	0.29(0.00)	0.00(0.00)	0.00(0.00)	0.00(0.00)	0.50(0.00)
	*BF Tree*	81.24(5.90)	0.79(0.07)	0.16(0.02)	0.79(0.08)	0.64(0.11)	0.70(0.08)	0.93(0.03)
	*Decision Stump*	19.34(0.68)	0.08(0.01)	0.28(0.00)	0.00(0.00)	0.00(0.00)	0.00(0.00)	0.54(0.04)
	*Hoeffding Tree*	14.41(0.09)	0.00(0.00)	0.29(0.00)	0.00(0.02)	0.00(0.02)	0.00(0.02)	0.50(0.02)
	*J48*	86.00(1.39)	0.84(0.02)	0.14(0.01)	0.85(0.05)	0.79(0.06)	0.82(0.04)	0.95(0.02)
	*LMT*	91.48(2.22)	0.91(0.02)	0.11(0.01)	0.95(0.05)	0.95(0.07)	0.95(0.06)	1.00(0.00)
	*NB Tree*	84.38(4.88)	0.83(0.05)	0.17(0.01)	0.72(0.09)	0.74(0.09)	0.73(0.08)	0.92(0.03)
	*Random Forest*	73.24(2.05)	0.70(0.02)	0.19(0.00)	0.54(0.06)	0.57(0.08)	0.55(0.06)	0.92(0.02)
	*Random Tree*	63.77(4.52)	0.60(0.05)	0.26(0.02)	0.46(0.09)	0.47(0.09)	0.46(0.08)	0.71(0.05)
	*REP Tree*	87.33(1.40)	0.86(0.02)	0.13(0.01)	0.83(0.07)	0.78(0.07)	0.80(0.05)	0.98(0.01)
Statistics-based	*Naïve Bayes*	59.04(3.66)	0.55(0.04)	0.23(0.01)	0.37(0.06)	0.49(0.09)	0.42(0.06)	0.83(0.04)
	*Bayes Net*	62.96(3.84)	0.59(0.04)	0.22(0.01)	0.44(0.07)	0.58(0.08)	0.50(0.06)	0.86(0.03)
	*Simple Logistic*	79.86(1.25)	0.78(0.01)	0.17(0.00)	0.70(0.09)	0.44(0.07)	0.53(0.07)	0.96(0.01)
	*Logistic*	99.34(0.97)	0.99(0.01)	0.04(0.01)	1.00(0.00)	1.00(0.00)	1.00(0.00)	1.00(0.00)
	*SMO*	18.06(0.99)	0.06(0.01)	0.28(0.00)	0.31(0.26)	0.03(0.02)	0.05(0.04)	0.63(0.04)
	*SVM*	16.33(0.52)	0.02(0.01)	0.39(0.00)	0.57(0.50)	0.02(0.02)	0.03(0.03)	0.51(0.01)
Perceptron-based	*MLP*	63.72(5.30)	0.60(0.06)	0.21(0.01)	0.60(0.20)	0.42(0.18)	0.44(0.11)	0.92(0.04)
Logic-based	*Decision Table*	73.48(3.20)	0.71(0.04)	0.20(0.01)	0.55(0.14)	0.66(0.12)	0.58(0.07)	0.94(0.02)
	*DTNB*	84.07(2.90)	0.82(0.03)	0.16(0.01)	0.78(0.08)	0.75(0.07)	0.76(0.05)	0.98(0.01)
	*JRip*	76.46(2.60)	0.74(0.03)	0.18(0.01)	0.90(0.09)	0.38(0.12)	0.52(0.12)	0.90(0.02)
	*PART*	86.64(1.49)	0.85(0.02)	0.14(0.01)	0.84(0.05)	0.80(0.07)	0.82(0.05)	0.95(0.03)
Instance-based	*IBK*	29.98(1.89)	0.22(0.02)	0.27(0.00)	0.25(0.05)	0.28(0.06)	0.26(0.06)	0.74(0.04)

Number of folds: 10, Type of experiment: cross validation, Number of repetitions: 10, Statistical test: paired T-test (corrected), Confidence: 0.01 (two tailed), SD: standard deviation

**Fig 5:**
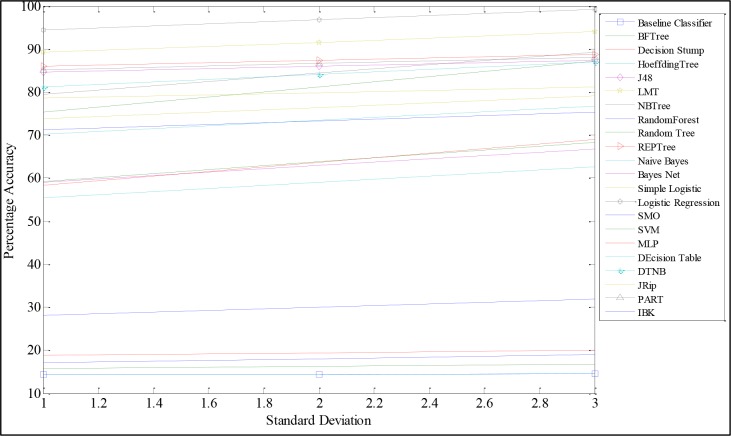
Comparison of Accuracy.

F-measure (0→1) measures the average rate of precision and recall of a classifier. It balances precision/recall tradeoffs. Kappa (Cohen’s kappa coefficient) statistics, on the other hand, measure the accuracy with respect to the p-value; thus, Kappa statistics measures the coincidental concordance between the output of a classifier and the label generation process. It compensates for random accuracy in a multi-class phenomenon. It ranges from -1 (total disagreement), through 0 (random agreement) to 1 (complete agreement), which implies that the computed accuracy depends on the efficiency and effectiveness of the classifier on the given observation. Our exploratory process, as shown Fig 5, revealed that some classifiers performed relatively better in distinguishing individual users. ZeroR, the baseline classifier, is the simplest form of classification and indicates the highest-class prior probability. It classifies all instances into one class using the modal frequency class. This study adopted the ZeroR baseline at the face value. However, given that ZeroR is a class prior probability-based classifier, we integrated a further baseline accuracy threshold of ≥ 90%. Six classifiers, as shown in Fig 5, met these criteria and were selected for further exploration. The six classifiers were the partial decision tree (PART), J48, REPTree, logistic model tree (LMT), Decision Table Naive Bayes (DTNB), and the logistic regression model. The logistic regression model uses a ridge estimator as the tuning parameter. Optimized result of the logistic model was obtained at the ridge estimator value of 2.0*e*^−6^.

As depicted in Fig 4, the dataset was resampled using a 70:30 sample size percentage without replacement, which represented the training and testing sets, respectively. The initial assumption would have been to adapt an ensemble classifier, such as bagging, to compensate for the repetition process in the overall instance size disparity between the original samples used in the experimental phase and the resampled instance. Given a dataset of *N* instances, the bagging algorithm builds a classifier by bootstrapping, whereby each sample has a (1−1N)N probability of selection, ≈ 0.36784, in this study. However, a comparative analysis of classification with and without the bagging algorithm resulted in a relatively similar output for the best classifier. Hence, we evaluated the training and testing samples without the Bagging. The results of the training and testing processes for the selected classifiers are presented in Table 10.

**Table 10:** Weighted Classification of Eleven Users.

Classifier	Evaluation metrics (weighted average)													
	*Accuracy %*		*RMSE*		*Precision*		*Recall*		*AUC*		*F-measure*		*Kappa stats*	
	*Train*	*Test*	*Train*	*Test*	*Train*	*Test*	*Train*	*Test*	*Train*	*Test*	*Train*	*Test*	*Train*	*Test*
PART	***94*.*37***	***84*.*53***	***0*.*08***	***0*.*03***	***0*.*98***	***0*.*85***	***0*.*99***	***0*.*85***	***1*.*00***	***0*.*82***	***0*.*99***	***0*.*85***	***0*.*94***	***0*.*83***
DTNB	67.39	65.77	0.21	0.21	0.70	0.68	0.67	0.66	0.94	0.93	0.67	0.65	0.64	0.62
LMT	**92.80**	**91.41**	**0.10**	**0.11**	**0.93**	**0.92**	**0.93**	**0.91**	**1.00**	**0.99**	**0.93**	**0.92**	**0.92**	**0.90**
J48	***95*.*00***	***84*.*53***	***0*.*08***	***0*.*16***	***0*.*95***	***0*.*85***	***0*.*95***	***0*.*85***	***1*.*00***	***0*.*95***	***0*.*95***	***0*.*85***	***0*.*94***	***0*.*83***
REPTree	89.17	84.37	0.12	0.14	0.89	0.85	0.89	0.84	1.00	0.98	0.89	0.85	0.88	0.83
**Logistic**	**99.68**	**99.31**	**0.041**	**0.05**	**1.00**	**0.99**	**1.00**	**0.99**	**1.00**	**1**	**1.00**	**0.99**	**1.00**	**0.99**

Testing instances: 1,887, Training instances: 4403

The logistic regression model with a ridge estimate value of 2*e*^−6^ performed better than the other classifiers. In terms of accuracy, the logistic regression model achieved 99.31% on the test set, which indicated consistency and effectiveness in distinguishing an individual based on the extracted features. Furthermore, other evaluation criteria, such as RMSE, Kappa statistics, and AUC, depicted the internal consistency and reliability of the model. Logistic regression model, models the posterior class probability (*G* = *j*|*X* = *x*) for J-classes through linear function, which produces linear boundary in instance space for different observed regions corresponding to different classes. This thus implies that the model developed by the logistic regression model is capable of providing a robust discriminating boundary for the given test sets. The logistic model tree (LMT) performed relatively closer to the logistic regression model with an accuracy of 91.41% on the test set. An LMT classifier is a hybrid classifier that integrates linear logistic regression model into decision tree (DT) classification mechanism. Classification is achieved by generating decisions with logistic models at its leaves, and prediction estimate is obtained by the use of posterior class probability. The integration of DT into LMT enhances its superiority over linear regression model when applied to a highly multidimensional dataset that requires ease of human interpretability. However, this dataset seems to exhibit quantifiable discriminating boundary, which favors linear logistic regression model.

J48 and PART performed relatively higher at the training stage with accuracies greater than 94%. However, the result of the test set fell below the 85% accuracy rate. This dissimilarity was also observed in the other evaluation criteria. The performance of DTNB classifier is inferior to logistic regression model and LMT in this study. DTNB is an integration of Naïve Bayes algorithm into decision table mechanism. An initial experiment based on Naïve Bayes shows a very poor classifier performance. Naïve Bayes classifier assumes that all attributes in the dataset are independent. The capability of LMT to infer larger structural knowledge from a high dimension dataset can be attributed to its superiority over DTNB.

PART is a rule-based induction algorithm which builds decision tree by avoiding global optimization in order to reduce the time and processing complexities. PART uses the separate-and-conquer approach of RIPPER and combines it with the decision tree mechanism of C4.5 by removing all instances from the training dataset that are covered by this rule and proceeds recursively until no instance in the dataset remains. PART builds a Partial decision tree for the current set of instances by choosing leafs with the largest coverage as the new rule. Moreover, logistic regression model and LMT demonstrated higher classification capability on the test sets than PART in this study.

A Reduced Error Pruning Tree (REPTree) applies regression tree logic and generates multiple trees in altered iterations by sorting values of the numeric attributes once. This is achieved through information gain principle (which measures the expected reduction in entropy), tree pruning based on reduced-error pruning with the back fitting method, and integration of C4.5 mechanism for missing value by splitting each corresponding instances into fractional instances. However, both logistic regression model and LMT demonstrated higher classification accuracy on the test sets than REPTree.

A J48 decision tree is a java coded version of C4.5 decision tree implemented in the WEKA workbench. C4.5 decision tree is an induction based learning algorithm, which uses information gain ratio (as oppose to ordinary information gain which is biased towards large value attributes) as splitting criteria for recursively partitioning instances of attributes into attribute-space. Classification of the instance is done by constructing nodes that form root tree using singular incoming edges to link nodes while supporting multiple outgoing edges through predefined discrete function of input attribute value. The performance of logistic regression model and LMT showed higher classification accuracy over J48 on the current test set. The performance of J48 on the training set is higher than that of LMT; however, the model built by LMT presents a more robust measure for discriminating users. Discussion on the reliability of the obtained result is presented in the next section

## Reliability of Observed Patterns

To evaluate the reliability of the observations presented in ‘Exploration Based on Classification of Individual user,’ we evaluated the expansion of the sample size of users considered in the classification process. The results in that section were achieved based on a sample size of 11 users and sample criteria of instance size of ≥ 300 sessions. In this section, the study considered the probability of obtaining reliable accuracy using much fewer variations in the sampling threshold. In [5,9,10], ten to fourteen users were considered with a session size ranging from 40 to 206 session instances. Furthermore, in [8], it was found that the instance size of ≥ 102 sessions is sufficient for a ‘click-print’ existential study. However, the assertion was contingent on the aggregation of sessions. Similarly, in [9], it was observed that the aggregation of web sessions could produce a significant accuracy improvement, experimental complexity notwithstanding. The current study thus considered a slight deviation from the logic of aggregation based on the assumption that the structural characteristics of individual browsing patterns are summarized by aggregation, which could reduce the observable dynamics in the request patterns of each user. This logic is also supported by the observation in [9] that non-aggregated user-centric features are sufficient for revealing ‘click prints.’ The user sample sizes defined in *D*_*is*_ and *T*_*is*_ are adapted for the reliability observation process.

$$D_{is}=U_{is\;}\times 2\;where\;U_{is}\;represents\;initial\;sample\;size$$

$$T_{is}=U_{is\;}\times 3\;where\;U_{is}\;represents\;initial\;sample\;size$$

A threshold-based sampling technique of a session size of ≥ 200 sessions and ≥ 100 sessions was adapted for *D*_*is*_ and *T*_*is*_, respectively. *D*_*is*_ and *T*_*is*_ represent double and triple sizes, respectively. The sampling of data based on a threshold of ≥ 200 session instances as the baseline resulted in 21 users, which is approximately equal to the logical definition in *D*_*is*_. Given the threshold of ≥ 100 sessions as the baseline, 31 users satisfied this criterion. This is approximately equal to the logical baseline defined in *T*_*is*_. The obtained experimental result for *D*_*is*_ and *T*_*is*_ is presented in Table 11.

**Table 11:** Training and Testing Analysis for Double and Triple Sample Sizes.

Scheme	Double Sample Size		Triple Sample Size	
	Training (%)	Testing (%)	Training (%)	Testing (%)
***Logistic***	***99*.*68***	***99*.*08***	***87*.*93***	***88*.*68***
DTNB	65.41	73.82	54.68	52.21
PART	77.60	77.79	75.87	75.77
LMT	83.61	85.38	75.08	76.29
J48	80.69	82.44	83.32	83.81
REPTree	77.19	80.63	80.05	82.62

*D*_*is*_ Testing instances: 2,819, *D*_*is*_ Training instances: 6,577, *T*_*is*_ Testing instances: 3,285, *T*_*is*_ Training instances: 7,664

The logistic regression model demonstrated a consistent class distinction among all other observed classifiers. A transition from the initial sample size to the double sample size reveals an overall improvement in the classification accuracy. In particular, three users (User-2, User-5, and User-11) exhibited significant improvements, as shown in Table 12. This suggests that a threshold of ≥ 200 session instances provided a reliable criterion for a ‘click print’ signature. However, a reduction in the classification accuracy of the triple sample size was observed. This prompted the supposition that the adapted threshold of ≥ 100 session instances is not sufficient to support monotonicity of accuracy. Monotonicity is defined in context as a linear relationship between the increase in sample size and observed accuracy. At face value, this supposition seems incongruent. However, a transition from the initial sample size to the triple sample size through the double sample size revealed patterns in the observed accuracy. A sample size increase of users with a threshold of ≥ 300 session instances showed the consistency of classification accuracy. A transition from a threshold of ≥ 200 session instances to threshold of ≥ 100 session instances recorded a relatively consistent accuracy, except for two users (User-16 and User-20), as shown in Table 12. A significant difference of 45.91% and 43.48% was observed in the accuracies of User-16 and User-20, respectively, given the transition from *D*_*is*_ to *T*_*is*_. The accuracy of the threshold of ≤ 200 session instances, which constituted the triple sample size, was shown to be significantly low. This suggests that session instances of ≤ 200 may not be sufficient to reveal an individual online ‘click print’ signature without cross-session aggregation.

**Table 12:** Accuracy of Logistic Regression Model for Different Sample Sizes.

User ID	Initial sample size (%)	*D*_*is*_ sample size (%)	*T*_*is*_ sample size (%)
User-1	100	100	99.52
User-10	100	100	100
User-11	**100**	**98.85**	**99**
User-2	**97.38**	**94.48**	**100**
User-3	100	100	100
User-4	99.62	100	99.17
User-5	**98.61**	**97.9**	**99.41**
User-6	100	100	100
User-7	100	99.08	99.02
User-8	100	100	99.61
User-9	100	100	100
User-19		100	100
User-21		97.06	96.49
User-15		100	100
User-18		100	96.59
***User-16***	** **	***97*.*76***	***51*.*85***
***User-20***	** **	***100***	***56*.*52***
User-14		100	97.22
User-13		97.86	100
User-17		94.59	100
User-12		100	100
User-27			38.18
User-28			36.58
User-26			29.79
User-24			33.33
User-29			31.11
User-23			48.48
User-30			25
User-22			56.1
User-31			22.64
User-25			32

### Observations of Exploration

**Fig 6:**
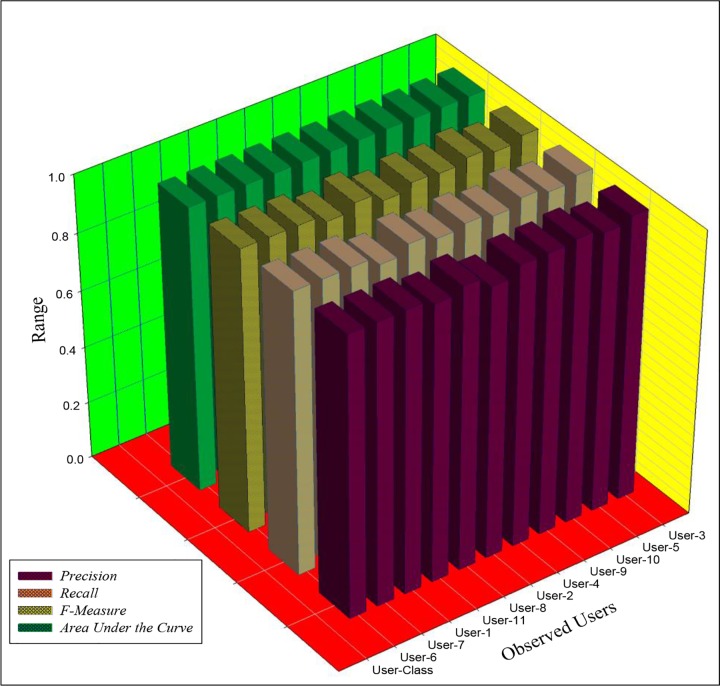
Metric of Evaluation of Testing Dataset.

As mentioned, the aim of this study is to explore the probability of individual uniqueness in online communication to determine if a reliable online identification process can be potentially harnessed. Most studies relating to user behaviors are concerned with network and/or application profiling and therefore present generic findings. The following observations were deduced from the empirical process conducted in this study:

1. With reference to Tables 6 and 7, a power law can closely model human online behavior. The duration of visitation patterns can be modeled with a power law, which has a varying model coefficient. This suggests a high probability of online identification based on the duration of visits within a predefined session boundary. Similarly, the number of requests per session obeys a generalized polynomial model. The observed power law model demonstrated a (dis)similar model coefficient. This observation further suggests the probability of an underlying factor that guides human interaction on the Internet. With reference to ‘Transformation of Inter-request Patterns,’ the study revealed a unique sequitur in online behavior. The observed sequiturs were independent of seasonal fluctuations, however; sequitur size varies across users. This further suggests consistency in the behavioral patterns of each individual.
2. With reference to the empirical observation in [4], in which a derived log-polynomial-exponent (LPE) function efficiently captures a long-tail feature of human browsing behavior, this study revealed that the observed LPE function has an equivalent power law. Furthermore, with reference to the empirical observation in [10], where the decay of visitation patterns correlates with the visitation pattern, this study revealed that the length of a session is significantly related to the visitation patterns and aggregated visitation patterns.
3. With reference to Table 10, online users can be distinctly identified. Further, feature selection revealed that the relationship between visitation patterns, session length, and aggregation of visitation patterns is principally responsible for individual distinction. These features provided better discriminatory information for online identification. This observation differs from the observation in [8], in which aggregation of sessions was adapted to observe user a ‘click print’ signature. The integration of additional informative and discriminative features presents a robust mechanism for online user identification technique. Furthermore, the observed accuracy showed that, with the application of a logistic regression model with an optimized ridge estimator, the online browsing sessional behavior of a user can be effectively distinguished.
4. In terms of data, in Fig 3, the study revealed that the higher the number of users under investigation, the higher the complexity of defining a cluster boundary to delineate different users. The finding further showed that an increase in data structure complexity is responsible for the poor performance of various clustering algorithms in online user distinction processes. The clustering algorithm performed better when there was a probable boundary hyperplane that separates the classes under observation. Therefore, given the poor boundary among the classes, it is logical to achieve poor clustering accuracy. Classification algorithm, on the other hand, performed better in extracting user dissimilarity. This can be attributed to the process of classification, which considers the relationship among discriminative features in a given feature space. Table 13 presents the results of the most reliable algorithm among the evaluated classifiers. The results clearly showed that each individual can be identified regardless of the number of users.
5. From the training results shown in Table 13, the logistic regression model achieved perfect accuracy for all users except for User-2 and User-5, on which the accuracy of 97.38% and 98.61%, respectively, was achieved. Similarly, the result of the F-measure of the training model showed that the logistic model achieved unity in distinguishing the individual user for all users except User-2 and User-5. The result from the testing set showed relative consistencies with the training set. The accuracy on User-11, User-2, and User-9 fell below 98% accuracy. Fig 6 presents a diagrammatic view of the result of the test set. The figure revealed the distinct similarity in the evaluation metrics.

**Table 13:** Result of Logistic Model with Ridge Estimate for Each User.

Class	*Training Result*				*Testing Result*			
	Performance Evaluation Metrics							
	Accuracy (%)	Precision	F-measure	AUC	Accuracy	Precision	F-measure	AUC
User1	100	1.00	1.00	1.00	100	1.00	1.00	1.00
user10	100	1.00	1.00	1.00	100	1.00	1.00	1.00
user11	100	1.00	1.00	1.00	***96*.*97***	0.98	***0*.*98***	1.00
user2	***97*.*38***	***0*.*985***	***0*.*979***	1.00	***97*.*89***	0.959	***0*.*97***	1.00
user3	100	1.00	1.00	1.00	100	1.00	1.00	1.00
user4	100	1.00	1.00	1.00	99.12	0.991	0.99	1.00
user5	***98*.*61***	***0*.*975***	***0*.*981***	1.00	98.2	0.982	0.98	1.00
user6	100	1.00	1.00	1.00	100	1.00	1.00	1.00
user7	100	1.00	1.00	1.00	100	1.00	1.00	1.00
user8	100	1.00	1.00	1.00	100	1.00	1.00	1.00
user9	100	1.00	1.00	1.00	***97*.*77***	0.99	0.98	1.00

### Comparative Analysis of Results

Observation of a singular sample-size unit revealed a higher accuracy than in results obtained in [5,9,10] and [32] The obtained result is also similar to the accuracy obtained in [8]. However, the accuracy in [8] was obtained at the fifty-first aggregation level and a uniform class prior probability of 10% for all observed users. The current study supports the assertion in [8] of an online ‘click print’ signature. Further, partial monotonicity was observed in this study, which is contrary to the complete monotonous observation in [8]. The result is thus similar to the observed monotonicity in [9]. Moreover, the observed limitation can be attributed to the relatively smaller number of users who have a threshold of ≥ 300 session instances. While fluctuation in accuracy was observed in the transition from the double sample size to the triple sample size, as shown in Table 13, it can be assumed that a larger sample size can be used to substantiate this assertion. The reliability of the observation portends stable classification accuracy of individual sampled-user. The comparative analysis presented in S1 Appendix showed the trend in an online attribution study (the S1 Data used for this study is given as supplementary file). Furthermore, it explicates the need for integration of online behavioral features, which can present a composite description for online attribution. The sample size considered in this study is consistent with existing studies, and the considered features integrate combinatorial features of tempo-spatial characteristics of humans. In terms of accuracy, the results showed similar performance to those of other studies, particularly the findings in [8]. Our exploration of consistency and introduction of a seasonal evaluation, uniquely distinguishes our study in relation to others. Furthermore, our exploration of multiple classifiers, although not exhaustive, provides a comparative analysis of various classifiers on online attribution and meaningful insight into online dynamics. The class prior probability considered in this study is not uniform. This yields the practical perspective on the online attribution process, as highlighted in [8], that online users may not necessarily have uniform class prior probability. A uniform class prior probability is therefore not a precursor to online user re-identification study since the presence of the behavioral pattern is not dependent on an equal number of sessions for each observed User.

### Research Limitations and Future Opportunities

As shown in Fig 6, the results of the classification process are not perfectly accurate, and the implication of inaccuracies in the online user identification process is cost sensitive. Cost sensitivity is described in this instance to refer to the probability of occurrence of false positive and false negative outputs, during the classification process. A higher probability of false positive/negative implies that a wrong User is identified. In an investigation, such wrongful identification could result in wrongful conviction. The number of human-centric features used in this study (15 features in total excluding the class variable), could be insufficient. Although visitation patterns were extracted, revisitation characteristics of users were not considered in this study. Revisitation pattern can be integrated into future study. This can provide insight into the interest of the user or relevance of the web page being visited by the user. Information such as web page demographics could be an additional source of discriminative features. These are potential features, as asserted in [10]. In addition, feature weighting and a feature selection algorithm were not applied to ascertain features with the most relevant weight, which could otherwise provide an optimal discriminative capability. While such a technique is beyond the scope of this study, a more robust classifier that considers feature and classifier optimization could be a potential source for a robust user re-identification process. As an ongoing research, the authors intend to further explore the probability of obtaining higher classification accuracy in re-identifying users, by adding the collection of a sufficient dataset for each user as well as increasing the sample size. A sample size of 31 users may not be able to provide a generalizable benchmark for an online user re-identification study. As asserted in [9], this could limit the generalizability of the present results, thus, a larger sample size could reveal higher variant characteristics among individuals. This study did not consider the exploration of the unique sequitur as either a stand-alone method for online user re-identification or an integrative composition for an online re-identification process. Text mining algorithms could be applied to the sequitur for an online user re-identification process.

## Conclusions

This paper presented a methodological assessment of online user attribution study. The study attempted to address two pertinent research questions on online attribution: Is user behavior consistent over the Internet? If it is, to what extent can the distinction be applied to distinguish online users? The dynamic characteristics of individual users were initially observed and subsequently applied to the exploration of the probability of the existence of consistent online browsing patterns using the fundamental unit of client-server communication processes. A consistency in patterns was observed through the discretization and subsequent symbolic transformation of the inter-request time series of individual users, which were partitioned based on seasonal factoring. The observed consistency in browsing behavior was then applied to observe individual (dis)similarity using multiple machine-learning classifiers. The obtained accuracy was subjected to a sample size robustness test. Logistic regression model was shown to produce more accurate and reliable classification results than the other classifiers. However, the logistic model tree and J48 decision tree also performed relatively higher. Furthermore, this paper presented a comparative analysis of research in the attribution of online users. The results showed that an individual user can be identified in a typical client-server communication based on a threshold of ≥ 200 session instances. This conforms to the findings in existing literature on online user attribution. The results further reveal a paradoxical characteristic in online communication patterns: on one hand, it reveals the probability of an individual request signature; on the other hand, it also reveals a high probability of individuals sharing a similar request signature. This was observed in the analysis of the symbolic transformation of individual request patterns. This finding revealed the need for a study that focuses on extracting group (dis)similarity in online communications. Such type of research could provide a better measure for a one-to-many identification process in online communications. In addition, a more robust technique can be applied to measure individual distinction, such as meta-classifiers. A meta-classifier can be applied to observe the probability of reducing the threshold of session instances. The integration of session aggregation, as computed in [9], can also be applied to improve the observed accuracy of the online attribution process.

## Supporting Information

S1 Appendix (DOCX)

S1 Data Dataset used in the machine learning process. (RAR)
