# Supplementary material for: Observing Consistency in Online Communication Patterns for User Re-Identification
Source: PLoS One. 2016 Dec 5;11(12):e0166930. doi: 10.1371/journal.pone.0166930 (PMC5137900; doi:10.1371/journal.pone.0166930)
Supplement: S1 Appendix — (DOCX) [file pone.0166930.s001.docx]

| **Author** | **Data Source** | **Features Adapted** | **Technique Adapted** | **Sample Size** | **Observed Accuracy** | **Additional Information** |
| --- | --- | --- | --- | --- | --- | --- |
| (1) | Web browsing history (client side) | Session duration, number of viewed pages, average time spent per page, day of the week | J4.8 classifier | 10 | 99.4% | Aggregation = 51, class prior = 10% Monotonicity observed |
| (2) |  |  | Heuristics based on Lift, support, and J48 pattern | 50 | 90.12% | Sliding window = 100, based on |
| (3) |  |  | J48 classifier | 50 | ≥ 90% | Monotonicity not observed. Aggregation varies from 1 to 30 |
| (4) | Click stream (client side) | Genre, days of week, time of day, number of page views, average duration of page views, time between page revisits | One class SVM, using ensemble classifier | 10 | 56% | Accuracy = (TP - TN) – (FP + FN) |
| (5) |  |  | Multiclass classifier | 12 | 75 -> 100% |  |
| (6) |  |  | Conditional random field with feature weighting learning | 14 | 66% ±7 | Accuracy = (TP - TN) – (FP + FN) |
| (7) |  |  | Hidden Markov model | 10 | ˂ 0.6 | Accuracy based on F-measure |
| (8) | DNS query (server side) | Session-based aggregation | Multinomial naive Bayes classifier with n-gram and cosine similarity | 2,100 | 88.25% | Actual number of sample size studied not discussed. |
| (9) |  |  | Multinomial naive Bayes | 28 | 73% | N/A |
| ***Current Study*** | ***Server side*** | ***Inter-request time series, session duration, moments of request, revisit pattern, number of request per session*** | ***Vocabulary based on sequitur and set theory, J48, logistic regression, DTNB, REPTree, PART, LMT*** | ***11*** | ***99.31%*** | ***The unique vocabulary that indicates high internal consistency in web browsing behavior of sampled users. Inter-Session Aggregation = 1, implies no aggregation is considered for each session.*** |
|  |  |  |  | ***21*** | ***99.08%*** |  |
|  |  |  |  | ***31*** | ***88.68%*** |  |

**APPENDIX A**

References

1. Padmanabhan B, Yang Y. Clickprints on the web: Are there signatures in web browsing data? Available SSRN http//ssrn.com/abstract=931057 or http//dx.doi.org/102139/ssrn931057 [Internet]. 2007 [cited 2014 Aug 25]; Available from: http://papers.ssrn.com/sol3/papers.cfm?abstract_id=931057

2. Yang YC. Web user behavioral profiling for user identification. Decis Support Syst. Elsevier; 2010;49(3):261–71.

3. Yang Y, Padmanabhan B. Toward user patterns for online security: Observation time and online user identification. Decis Support Syst [Internet]. 2010 [cited 2014 Mar 30];48(4):548--558. Available from: http://www.sciencedirect.com/science/article/pii/S0167923609002255

4. Abramson M, Aha D. User Authentication from Web Browsing Behavior. In: The Twenty-Sixth International FLAIRS Conference [Internet]. Florida: AAAI DIGITAL LIBRARY; 2013 [cited 2014 Aug 25]. p. 268–73. Available from: http://www.aaai.org/ocs/index.php/FLAIRS/FLAIRS13/paper/viewFile/5865/6081

5. Abramson M, Gore S. Associative Patterns of Web Browsing Behavior [Internet]. 2013 AAAI Fall Symposium Series. Palo Alto, California; 2013 [cited 2014 Aug 25]. Available from: http://www.aaai.org/ocs/index.php/FSS/FSS13/paper/viewPDFInterstitial/7587/7560

6. Abramson M. Learning Temporal User Profiles of Web Browsing Behavior. Nav Res Lab [Internet]. 2014 [cited 2014 Dec 18];1–9. Available from: http://www.nrl.navy.mil/itd/imda/sites/www.nrl.navy.mil.itd.imda/files/files/LearningUserProfilesSocialCom-live.pdf

7. Abramson M. Toward the attribution of web behavior. In: In Computational Intelligence for Security and Defence Applications (CISDA), 2012 IEEE Symposium [Internet]. Ottawa, Canada: IEEE; 2012 [cited 2014 Aug 25]. p. 1–5. Available from: http://ieeexplore.ieee.org/xpls/abs_all.jsp?arnumber=6291524

8. Banse C, Herrmann D, Federrath H. Tracking users on the internet with behavioral patterns: Evaluation of its practical feasibility. Inf Secur Priv Res Springer Berlin Heidelberg [Internet]. 2012 [cited 2014 Dec 8];IFIP AICT:235–48. Available from: http://link.springer.com/chapter/10.1007/978-3-642-30436-1_20

9. Herrmann D, Gerber C, Banse C, Federrath H. Analyzing characteristic host access patterns for re-identification of web user sessions. In: Editors: Tuomas Aura, Kimmo Järvinen KN, editor. Lecture Notes in Computer Science (including subseries Lecture Notes in Artificial Intelligence and Lecture Notes in Bioinformatics) [Internet]. ISBN: 978-. Espoo, Finland: Springer; 2012 [cited 2014 Dec 8]. p. 136–54. Available from: http://link.springer.com/chapter/10.1007/978-3-642-27937-9_10
